# On the Willingness to Report and the Consequences of Reporting Research Misconduct: The Role of Power Relations

**DOI:** 10.1007/s11948-020-00202-8

**Published:** 2020-02-26

**Authors:** Serge P. J. M. Horbach, Eric Breit, Willem Halffman, Svenn-Erik Mamelund

**Affiliations:** 1 Faculty of Science - Institute for Science in Society, Radboud University Nijmegen, P.O. Box 9010, 6500 GL Nijmegen, The Netherlands; 2 Center for Science and Technology Studies, Leiden University, Leiden, The Netherlands; 3 Work Research Institute, OsloMet – Oslo Metropolitan University, St. Olavs Plass, P.O. Box 4, 0130 Oslo, Norway

**Keywords:** Research integrity, Research misconduct, Whistleblowing, Power relations, Organisations

## Abstract

While attention to research integrity has been growing over the past decades, the processes of signalling and denouncing cases of research misconduct remain largely unstudied. In this article, we develop a theoretically and empirically informed understanding of the causes and consequences of reporting research misconduct in terms of power relations. We study the reporting process based on a multinational survey at eight European universities (N = 1126). Using qualitative data that witnesses of research misconduct or of questionable research practices provided, we aim to examine actors’ rationales for reporting and not reporting misconduct, how they report it and the perceived consequences of reporting. In particular we study how research seniority, the temporality of work appointments, and gender could impact the likelihood of cases being reported and of reporting leading to constructive organisational changes. Our findings suggest that these aspects of power relations play a role in the reporting of research misconduct. Our analysis contributes to a better understanding of research misconduct in an academic context. Specifically, we elucidate the processes that affect researchers’ ability and willingness to report research misconduct, and the likelihood of universities taking action. Based on our findings, we outline specific propositions that future research can test as well as provide recommendations for policy improvement.

**Electronic supplementary material:**

The online version of this article (10.1007/s11948-020-00202-8) contains supplementary material, which is available to authorized users.

## Introduction

In recent years, the attention paid to research integrity and misconduct has increased. Besides attempting to measure or estimate the extent of misconduct, several scholars also have investigated its causes. This literature shows that important drivers of misconduct range from individual personality traits to systemic factors, which include productivity pressure and corporate influences (Fanelli et al. 2015; Tijdink et al. 2016; Horbach and Halffman 2019). Much less attention has been paid to how scientific misconduct is detected and denounced, for instance, through the peer review system (Guston 2007; LaFollette 1992) or social control mechanisms such as whistleblowing (Stroebe et al. 2012). These processes are crucial for signalling misconduct and articulating what the research community deems un/acceptable behaviour. In addition, the detection and sanctioning of research misconduct depend almost entirely on discovery and reporting by peers, with the potential exception of plagiarism and some forms of statistical manipulation, which may be discovered through automated detection by means of ‘scanners’.

In this paper, we aim to provide a more elaborate theoretical and empirical understanding of the causes and consequences of reporting research misconduct. We do so by approaching the issue from a whistleblowing perspective (Near and Miceli 2016; Santoro and Kumar 2018; Vandekerckhove 2016) and by applying theories of power and power differences. The term ‘whistleblowing’ typically refers to distinct activities of informing authorities or the public that the organization one is working for is doing something immoral or illegal. In our use of the term, we include also ‘softer’ forms of reporting, such as talking to colleagues or one’s supervisor. To avoid confusion, we use the term ‘reporting’ and only use ‘whistleblowing’ in reference to literature explicitly using this term.

A better understanding of research misconduct reporting could contribute to improved early warnings and more effective preventive policies to promote research integrity. Rather than appealing to individuals to take responsibility and relying on sanctions to keep them in line, such policies should pay more attention to social processes, such as power imbalances, group pressure and performance pressure. More specifically, appropriate reporting policies should target a culture of complacency and cynicism that normalises questionable research practices, or even outright misconduct (Clair 2015; Martinson et al. 2010). The literature on organisational integrity in general has focussed on power imbalances, retribution concerns and career consequences (Bowie 2010; Palazzo 2007), which probably also play a role in research integrity.

In this study, we aim to better understand the processes that facilitate or inhibit the reporting of alleged research misconduct by analysing a large sample of direct and indirect witnesses of research misbehaviour. We draw on qualitative responses from a survey conducted in eight European academic research universities in 2017. In this survey, respondents who indicated they had directly or indirectly witnessed an instance of misconduct were asked to respond to open-ended questions about this instance and how they handled it.

We focus on the following research question: How do varying power positions influence the reporting, or not reporting, of alleged research misconduct? Given the data available to us, we focus on three specific power positions: academic seniority (i.e. the formal work position), work contracts (i.e. permanent vs. temporary appointments) and gender. These power elements have been identified as key factors in most commercial organisations’ studies on organisational integrity (Dozier and Miceli 1985; Cassematis and Wortley 2013; Culiberg and Mihelic 2017). We also study the influence of the specific type of misconduct, i.e. whether it involves a clear-cut type, such as plagiarism, or a more contested ‘questionable research practice’ (QRP), such as the disputed attribution of authorship.

To our knowledge, this is the first systematic study of researchers’ reasons for and accounts of reporting or not reporting witnessed misconduct. Our aim is to extend studies of ‘whistleblowing’ and of misconduct reporting from a predominantly corporate setting to that of academia. Our article is structured as follows: the “Literature” section presents an overview of the literature on research integrity and misconduct. In the “Theoretical Framework” section, we introduce studies of reporting and power, deriving factors potentially affecting researchers’ willingness to and the consequences of reporting alleged misconduct. The “Methods” section describes our study’s survey methodology, while  the “Results” section presents this survey’s main qualitative empirical results. In the “Discussion” section, these findings are formulated as propositions relating power relations to the reporting of alleged misconduct. We suggest these propositions can be used to explore further hypothesis-testing research on the relationship between power differences and misconduct reporting. Finally, the “Conclusion and Recommendations” section offers concluding remarks and policy recommendations.

## Literature

Over the past decades, research misconduct has drawn the attention of scholars from various fields. Currently, an extensive literature focusses by and large on the prevalence and causes of misconduct, or on questionable research practices (QRP). The literature focuses to a lesser extent on the consequences of misconduct, for example, how retracted journal articles affect careers (Azoulay et al. 2017), how institutions deal with alleged misconduct cases (Horbach et al. 2018), the consequences for research reliability (Horbach and Halffman 2017; Al-Marzouki et al. 2005), and on the role of scientific misconduct in general (Schulz et al. 2016).

This literature has also developed an inventory of ‘novel’ forms of academic misbehaviour (Callaway 2015; Sacco et al. 2018; Biagioli et al. 2019; Bouter et al. 2016), including estimations of how often some of these forms occur (e.g. Hopp and Hoover 2017; Fanelli 2009). Several authors highlight ‘risk categories’, including scientific fields and geographical areas where research misconduct or QRPs occur more frequently (e.g. Fanelli et al. 2015; Yang 2013; Stitzel et al. 2018). Similarly, the literature has outlined several potential causes of misbehaviour in science, including individual researchers’ personality traits (Tijdink et al. 2016); the organisational context in which these researchers operate (Anderson et al. 2007; Forsberg et al. 2018); and more systemic causes, such as competitive research funding and ‘publish or perish’ pressures (Fanelli et al. 2017; Sarewitz 2016).

Much less attention has been paid to the mechanisms that might detect and identify scientific misconduct. Some have argued that the peer review system is a prime example of such a mechanism (Guston 2007; LaFollette 1992), others’ hopes rest on social control mechanisms, most notably whistle-blowers and the close colleagues of misbehaving scientists (Stroebe et al. 2012). Despite the limited evidence, misconduct case studies suggest that alleged culprits’ close colleagues and peers are the most likely way of bringing misconduct to light (Horbach et al. 2018).

Specifically, the processes involved in signalling and reporting alleged misconduct are not well researched. For example, we are not aware of any study examining researchers’ motivations for reporting alleged academic misconduct, or of any research on such actions’ effectiveness. However, some studies have made a case for establishing ‘safe whistleblowing procedures in academic organisations’ (Forsberg et al. 2018). There is also a significant literature on whistleblowing procedures in the business ethics and management fields (e.g. Culiberg and Mihelic 2017; Palazzo 2007; Vandekerckhove 2016). Nevertheless, little is known about how these processes are applied in academic research institutions such as universities. Questions about who are most likely to report, their motivations for doing so, and the effectiveness and potential negative consequences of reporting remain unanswered. A deeper understanding of how and why researchers raise concerns or keep quiet can contribute to developing organisational conditions that will support a stronger culture of research integrity.

## Theoretical Framework

In addition to the literature on research integrity, there is a vast body of research on integrity in organisations in general, based on studies of wrongdoing in (or by) organisations (e.g. Palmer 2012), and research on organisational integrity management (e.g. Paine 1994). The literature on organisational integrity has paid more explicit attention to whistleblowing and the reporting of misbehaviour (Vandekerckhove 2016; Near and Miceli 2016). Among the more central theoretical questions in the literature on whistleblowing are the factors influencing (a) a witness of wrongdoing’s decision whether or not to report such instances; (b) the extent to which a reporter faces, or fears, retaliation; and (c) reporting’s effectiveness in terms of addressing wrongdoing. Besides the likelihood and effectiveness of reporting, the literature has also highlighted whistleblowing’s potentially negative consequences, such as psychosocial and reputational consequences (Bjørkelo and Matthiesen 2012; Culiberg and Mihelic 2017; Park and Lewis 2018).

The role of power relations is widely acknowledged in respect of whistleblowing or the reporting of wrongdoing in organisations. We therefore highlight two central conceptualisations of power in this literature. As theorised in the resource dependence theory, the first notion understands power as a central resource or asset that an individual may possess (Lukes 2005). From this perspective, an organisation’s less powerful members—such as younger employees, people with temporary work contracts, women, or people lower in the organisation’s hierarchy—are less likely to report alleged misconduct.

Several factors contribute to this decreased reporting likelihood. For example, these actors may have less access to powerful social networks in the organisation and therefore have less social capital. In addition, younger, and thus less experienced employees, may have less knowledge of the procedures and of how these are applied in practice. Low-resource members may also fear more retaliation and are generally less able to achieve genuine and desirable change as a result of reporting a case, such as adequate intervention in wrongdoing cases, or even improved integrity policies (Gao et al. 2015). This is especially true in cases involving more powerful wrongdoers.

A second theoretical dimension is French and Raven’s theory of social power, i.e. having the ability or being in a position to influence others. The theory involves five power bases: legitimate power (based on the legitimate right to prescribe behaviour), referent power (based on identification with one another), expert power (based on special knowledge or expertise), reward power (based on the ability to award resources), or coercive power (based on threats of punishment) (French et al. 1959). This suggests that reporters of alleged misconduct lacking such power bases are less likely to be effective, especially when reporting the misbehaviour of more powerful organisation members. This lack of power may affect the likelihood that they will report misconduct and the outcome of their reporting.

Based on the above, we expect researchers with fewer resources to be less likely to report alleged misconduct and their reporting to be less likely to result in effective interventions (Mesmer-Magnus and Viswesvaran 2005; Gao et al. 2015). Such researchers may include those in junior positions, such as doctoral students and post-docs, as well as those with temporary contracts. In addition, social power theory indicates that researchers with higher seniority (i.e. who have worked in academia longer) are more likely to (effectively) report misconduct cases (Cassematis and Wortley 2013; Gao et al. 2015).

Consequently, even though the role of power in whistleblowing has not been studied in an academic context, the variables (1) academic seniority and (2) temporal versus permanent work appointments are expected to affect the willingness to and the consequences of reporting research misconduct. In addition, some of the obstacles to and the potential consequences of reporting are believed to affect women more, although the evidence for this from the general integrity literature is inconclusive (Mesmer-Magnus and Viswesvaran 2005). To shed more light on the topic, gender will be explored as a third variable potentially influencing reporting.

Lastly, several studies have outlined the influence of the type of wrongdoing on the likelihood of reporting. In particular, clear-cut instances of misbehaviour are more likely to be reported than nuanced cases, which may be prone to different interpretations and normative assessments (Near and Miceli 1985; Mesmer-Magnus and Viswesvaran 2005). Accordingly, more indisputable forms of research misconduct, such as fabrication, falsification and plagiarism (FFP), are more likely to be reported than more questionable research practices, such as disputes over authorship or text recycling. Furthermore, if witnesses of wrongdoing perceive that there is high probability of their complaint being taken seriously and it is less likely to backfire, they are more likely to report it. We also study the type of witnessed misconduct’s influence as an additional variable in our model.

## Methods

### Data Collection

Data on research misconduct as a workplace issue were collected by means of a web-based, cross-sectional survey (Questback). The questionnaire is included as supplementary material. Central themes in this survey were organisational policies regarding misconduct and integrity, reporting mechanisms and attitudes, tensions arising from and the risks of research misconduct, perceptions of integrity measures, and the prevalence of research misconduct (Mamelund et al. 2018).

In this paper, we draw on data collected as part of the survey, which consisted of open-ended questions on the respondents’ possible first-hand knowledge of a research misconduct incident. This approach was adopted from the validated and revised Scientific Misconduct Questionnaire (SMQ-R) (Habermann et al. 2010; Broome et al. 2005). The open-ended questions were:

- How did you first learn about the instance of research misconduct?
- Please describe the specific instance of research misconduct.
- What did you do when you became aware of it?
- Whom (titles only) did you talk to?
- Were you able to talk to the individuals who were involved?
- Was the instance reported? To whom and by whom?
- What was the outcome? How did you feel about how it was handled?
- Did you think anything changed as a result?
- Is there anything you would have done differently?

#### Participant Selection

The survey was conducted among the employees of the European PRINTEGER project’s eight partner universities (PRINTEGER 2016). A PRINTEGER member sent a link to the survey questionnaire to the principal investigators in each of the partner universities. These investigators subsequently forwarded information about the survey and the link to a senior manager at their institution, who distributed these to the target population at their universities, i.e. the academic staff, excluding the technical and administrative staff. The reason for this distributed approach was to ensure a high response rate. The senior managers were encouraged to inform their academic staff of the survey and to highlight its importance.

Data collection took place from 7 March to 1 August 2017. The total population across the eight partner institutes comprises 20,815 academic staff members. Overall, 1126 respondents participated in the survey, with 194 responding to the open-ended questions. The demographical characteristics of the open-ended questions’ and the survey’s respondents are provided in Table 1. Table 1 provides information on the potential reporting bias by indicating the differences between the qualitative sample’s respondents and the participating universities’ general population. As shown in the table, the qualitative sample consists of older researchers and those with more senior positions (i.e. professors and associate professors) than found in the general population. The qualitative sample also consists of a higher proportion of social and behavioural sciences’ researchers.

**Table 1:** Demographic distribution of responses

Variables	Subgroups	Qualitative sample (%)	Survey sample (%)	Population (%)	Difference between qualitative sample and population
Gender	Male	52.7	47.8	53.6	− 0.9
	Female	47.3	52.2	46.4	0.9
Age	20–29	12.6	20.8	24.6	− 12
	30–39	27.8	30.5	32.7	− 4.9
	40–49	20.4	21.2	18.8	1.6
	50–59	24.0	16.2	15.4	8.6
	60 +	15.2	11.3	8.6	6.6
Position	Professor	31.9	18.1	12.2	19.7
	Associate professor	33.5	36.4	28.7	4.8
	Teacher	0.0	1.3	5.7	− 5.7
	Post-doc	12.4	10.6	22.5	− 10.1
	Ph.D. student/TA	22.2	33.5	30.9	− 8.7
Appointment	Temporary	45.0	51.0	NA	–
	Permanent	55.0	49.0	NA	–
Academic field	Engineering	5.3	5.0	5.3	0.0
	Language, info, comm	2.6	3.8	5.7	− 3.1
	Law, arts, humanities	15.3	17.1	20.1	− 4.8
	Medical/life science	27.4	23.6	27.7	− 0.3
	Natural sciences	21.0	17.3	24.7	− 3.7
	Social and behavioural	28.4	33.2	16.6	11.8

Each of the eight universities provided population data. Two challenges arose from the population analyses. Firstly, the coding system across the universities revealed inconsistencies regarding academic positions, especially regarding the meaning of ‘teacher’ and ‘academic field’. Where available, we used individual-level data and discarded the faculty information. Secondly, not all universities had access to staff members to cover all the demographic variables. For example, one of the universities could not reach its Ph.D. students.

#### Privacy and Ethics Approval

The relevant ethics committees at each participating institution granted their ethics approval of the survey. The privacy policy was explained before the participants started the survey and they were asked to agree to this, thus providing informed consent. The responses were collected anonymously and are not traceable to the respondents’ institutes. We present quotes from the responses with the demographic information that the relevant respondent provided.

### Data Analysis

The analysis used for this paper is explorative and inductive, i.e. we sought to develop hypotheses rather than to test them (Silverman 2016). Owing to the relatively small sample size and the diversity of the universities studied, as well as the richness of the qualitative responses to the open-ended questions, we refrain from an in-depth statistical analysis, focussing instead on the content of the open-ended questions’ responses. We thus explore the issues raised in the responses and develop propositions that future research can test. The relatively high number of qualitative responses allowed us to examine the different responses’ frequency to demonstrate more and less common responses, but not to estimate precise rates in terms of the population. Consequently, we do not provide significance levels and do not claim that these proportions can be generalised.

Second, we collapsed the nine initial questions (see above) into the following five categories due to the overlap between some of the questions: (1) type of research misconduct witnessed, (2) source that led to awareness of the misconduct, (3) initial reaction to the awareness, (4) type of reporting and (5) outcome of the reporting. Broome et al. (2005) took a similar approach, although the final categories are not entirely similar.

Third, we systematized the responses within each of the five categories by searching for patterns between the responses. We took an open coding approach, which meant that we assigned our own labels to the responses, rather than using a pre-existing template (Strauss and Corbin 1990). An exception was made in the ‘type of misconduct witnessed’ category, in which we used existing categorizations of misconduct, such as falsification, fabrication and plagiarism. We thus categorised responses into codes. At times, responses were categorized into multiple codes; consequently, the frequency of the codes may be larger than the overall number of respondents. This process of categorizing codes was iterative, i.e., some codes were collapsed and new ones developed as the analysis proceeded. “Appendix” provides an overview of the coding and the responses, including illustrative examples.

Fourth, once we had developed an appropriate selection of categories of the responses, we moved on to analyse the relationships between the responses and the demographical variables. We performed this analysis using simple cross-tables, i.e. based on counting the code frequency related to the different demographic variables. Given our theoretical interest in power relations, we focused especially on gender, age, academic seniority and work appointment. We also included type of misconduct in the analysis, distinguishing between clear-cut types of research misconduct and more nuanced forms of misconduct.

The results of these analyses are provided in the following sections. We present both results of the qualitative and the quantitative aspects, but the latter mainly serve as a contextualisation of the qualitative material forming the core of our results.

## Results

In this section, we will first (“Effect of Power Relations on Reporting” section) look more closely at the distribution of the responses with respect to the three variables outlined in Sect. 3: seniority, work contracts and gender. For each variable, we first present a brief overview of the quantitative distribution of responses related to respondents’ demographic information, after which we present and analyse the responses more qualitatively. We analyse the responses in terms of each variable in three ways: (1) whether or not a case was reported, (2) how and to whom a case was reported, and (3) the respondent’s perception of the consequences of reporting. We then (“Type Of Misconduct Reported” section) explore in detail how the respondents’ reporting varies across the different types of misconduct witnessed. These findings suggest how elements of power are involved in misconduct and its potential reporting, which lead to several propositions on power relations’ role in the reporting of alleged misconduct in “Discussion” section.

### Effect of Power Relations on Reporting

#### Academic Seniority

The existing literature on reporting suggests that researchers in junior or lower academic positions are less likely to report alleged misconduct compared to those in more senior positions. In our data, there is indeed a division between professors and researchers in lower positions. In our limited sample, professors reported a witnessed case of alleged misconduct more often (67% reported vs 29% not reported) than other members of academia, such as associate professors (37% vs 53%), post docs (35% vs 61%) and Ph.D. students or TAs (39% vs 51%). Since we defined reporting as giving account of a case to any party tasked with handling such cases, these observations hold even when taking into consideration that some junior researchers would report misconduct to senior researchers, who would then report it to official channels, such as a research integrity committee.

When we examine the responses across age groups, more senior researchers are also more likely to report misconduct. While respondents in the age group 20–29 claim to have reported misconduct in 33% of the witnessed instances, the other age groups’ percentages are 32% (30–39), 51% (40–49), 65% (50–59), and 51% (60 +). In this small set, the effect of age and academic rank could not be isolated, but it does suggest that age should currently not be ignored as a significant factor.

The qualitative responses provide more insight into the reasons for reporting or not. Notions of power, in this case in the sense of resource availability, seem to play a crucial role. A prominent reason for junior researchers not to report was their fear of negative consequences, such as losing future opportunities or the hampering of their social relations at work. This is exemplified in the following quotes:

No, I did not push it further for fear of career consequences (30-39, female, Law/Arts/Humanities, PhD student, temporary, 0-5 years).

I reported to the direct supervisor, but was sure I could not go beyond that, as that would directly have impeded my own relation with my own supervisor (I am a PhD student) (20-29, male, Natural Sciences, PhD student, temporary, 0-5 years).

No, I decided to not report it, because I'm a junior researcher and afraid that it would affect my career possibilities (20-29, female, Medical/Life Sciences, PhD student, temporary, 0-5 years).

Even though less common, some more senior researchers also reported this fear of negative consequences:

Nothing. If I said sth., I'd be disadvantaged in all [aspects] of my work. Office politics (No age, no gender, Natural Sciences, permanent, assistant/associate prof, 16+ years).

Another frequently mentioned reason was distrust of the management’s willingness to take any corrective action, as exemplified below:

No. My department manager never takes any action on any problem (40-49, female, Social/Behavioural Sciences, PhD student, temporary, 0-5 years).

A third reason mentioned is a belief that reporting would not lead to any changes, notably due to certain individuals being protected. These responses show how issues of seniority, hierarchy and power affected respondents’ decision not to report an alleged case:

No. My old boss is too powerful in the community (30-39, male, Natural Sciences, leadership role, assistant/associate prof, temporary, 6-10 years).

Nothing, there is no going against my boss. There have even been lawsuits in the past, but the University has always covered for her (30-39, female, Law, Arts and Humanities, PhD student, temporary, 0-5 years).

In all these examples, the common hierarchical structure in academia plays a prominent role in an actor’s decision to report or not. Both conceptions of power outlined in section three (resource dependence and French and Raven’s theory of social power) become visible in the respondents’ comments. Specifically, the control of resources, such as senior colleagues’ research and promotion opportunities, seems to be a prime concern. The latter even extends beyond the research organisation’s immediate environment to the wider research field and peer community. The organisational and wider research context may thus be a source of normalising behaviour – among others due to restricted reporting.

Another response mentioned that power relations, in particular seniority, may not only affect the reporting of alleged misbehaviour, but may actually be one of its causes. A female professor in the life sciences explains how she was ‘pressured’ into behaving in dubious ways:

I was working with a more senior professor to promote findings of some research through a prestigious impact/ knowledge mobilisation event which was presenting the 'best evidence to inform practice'. The prof wanted to promote a tool we had developed as having a positive impact. Myself and the wider research team had concerns that we had no evidence of the tool's efficacy and in fact the small feasibility study had raised some concerns about its effect. We wanted to wait until the full trial was complete before promoting it as a tool. Although we had agreed as a team the limits of what could be said about the tool, I was the only team member working with the professor on this impact event and a few days before, he called me to [participate in] a teleconference with the sponsor of the event, and they both put a lot of pressure on me to allow the tool to be presented as effective. When I started to explain to the sponsor what the concerns of the team were, the professor muted the call and told me not to tell her that! It was a very intimidating situation and I felt I had to withdraw (40-49, female, Medical/Life Sciences, leadership role, assistant/associate prof, permanent, 16+ years).

The mentioned fear does not only concern fear of superiors, but also fear—especially related to such superiors—of research misconduct becoming public. Thus, while power plays a key role in discouraging reporting, senior researchers may not perceive their wielded power as self-serving, but as an effort to protect a collective interest, often the research institution’s image (although this could be considered misguided). The following quotation is from a female Ph.D. student explaining why she did not report an incident after consultation with her professor:

Only to the professor, who insisted it [should] not [be] reported to the ethics officer. They didn't want it to become public and wanted to fix it themselves (20-29, female, Medical and Life Sciences, PhD student, temporary, 0-5 years).

The following is a similar response from a more senior respondent:

The instance was not reported to maintain the reputation of the faculty. The decision was made solely by the professor involved (60-69, female, Natural Sciences, researcher, permanent).

We also examined to whom the incidents were reported. Our data suggest that the different levels of seniority have different reactions. Of our respondents, professors (26%) and associate professors (27%) informed a supervisor more often about an incident than Ph.D. students/TAs (12%) and postdocs (4%). Conversely, Ph.D. students/TAs (29%) and postdocs (35%) responded more often by talking to their colleagues about the misconduct than professors (7%) and assistant/associate professors (11%) did. Furthermore, professors and associate professors confronted the culprits more often (26% and 24%) than postdocs (17%) and Ph.D. students/TAs (12%). Although the tendency is not very strong, this may be cautiously interpreted as ‘soft’ responses dominating with regard to junior researchers, while more senior academics make use of ‘harder’ means.

Our material also showed that respondents in more senior positions more often perceived the outcome of reporting alleged misconduct as constructive, than those in junior ones. Professors experienced a constructive change (34%) most often, followed by associate professors (23%), postdocs (22%) and Ph.D. students/TAs (17%). The tendency is similar across ages. Overall, this level of reporting indicates that a perceived constructive change is uncommon across all positions, but even more so regarding the most junior academic positions; however, the limited observation size indicates that the findings should be regarded with caution.

The following responses by a female Ph.D. student are an example of ‘no change’ after reporting an incident. She reported an issue of undeserved authorship, primarily due to work-related frustrations:

To [an] ombudsperson by me (PhD student). Decision was made because I was suffering greatly as a PhD student under her (sic.) supervisor; several other instances of misconduct also applied in our work relationship, in addition to emotional abuse (being personally criticized, being called an[d] yelled at after hours, being pressured into misconduct, ...) (20-29, female, Social and Behavioural Sciences, PhD student, temporary, 0-5 years).

She continued to explain that reporting her supervisor was difficult due to the latter’s social position and, crucially, that she had refrained from reporting any misconduct ever since:

The attitude seemed to be that as a senior she [had to] know what she was doing, and as a junior researcher, I felt met with disbelief. Little action was undertaken, and I have refrained from reporting any misconduct ever since.

The expectation that action will be taken and the perceived guarantee that reporting a case will have an effective outcome can increase academics’ willingness to report cases. Organisational procedures, such as reporting to an ombudsman, could offer powerful resources to redress imbalances, but these have to provide convincing intervention opportunities. Our data indicate that building perceptions of adequate handling into procedures positively affects respondents’ willingness to report.

Finally, the issue of power was not always mentioned as a barrier to reporting others’ misconduct. Our material also had an example of a Ph.D. student who had benefited from her supervisor’s power use. This student used the survey to reflect on this incident:

I talked with the professor, who put my name on the paper; he felt he had done me a favour and I did not object. Not reported. I decided it was good for my career and I should just leave it (regarding a case where “my name was put on a paper that I had had nothing to do with”; 20-29, female, Medical/Life Science, PhD student, temporary, 0-5 years).

#### Work Appointment

The second variable we explored constitutes the relation between employment precarity and misconduct reporting. This was based on the assumption that researchers with temporary contracts are less likely to report alleged misconduct compared to researchers with permanent contracts. As we will show, our data suggest that the employment conditions and academic seniority patterns are similar. A confounding factor between the two variables could be that, in general, professors and associate/assistant professors are likely to have permanent positions, while PhD student, teaching assistant and Post-doc positions are usually, if not always, temporary.

Examining the answers to the respondents’ willingness to report, we found that researchers in permanent positions report incidences of suspected misconduct twice as often as those in temporary positions. Whereas 59% of researchers in permanent appointments reported such incidence, only 31% of those in temporary appointments did so.

In the qualitative responses, the respondents never explicitly mentioned temporal employment, which was only indirectly mentioned in their responses through references to power and hierarchy. For example, a common reason for researchers in temporary positions not reporting was their fear of negative career effects. The following quotation exemplifies this fear:

No, I can't because of hierarchy. It is a superior (sic) and denouncing could have a negative impact on my job (40-49, gender: other, Law, Arts and Humanities, assist/assoc prof, temporary, 11-15 years).

No, because this study was important [for] the dissertation of the PhD student who had limited time. I didn't feel like I had enough support to get out of this unharmed (male, 30-39, left academia, temporary, Social Sciences).

Others attributed their lack of reporting to the management not taking them seriously:

I did not report this explicit situation. I have however gone to the ombudsperson for similar situations (p-hacking, unauthorized authorship, ...) and was again discarded (sic) as […] the junior one with no experience (20-29, female, Social Sciences, PhD student, temporary, 0-5 years)

Such responses and ways of reasoning were far more common in respect of respondents with temporary work contracts compared to their permanently appointed colleagues. The same holds for another common response, namely a lack of knowledge regarding what to do, i.e. where and how to report:

No, I wasn't directly working with him anymore when I found out and I didn't know what to do (30-39, female, Social and Behavioural Sciences, assistant/associate prof, temporary, 11-15 years).

No, no idea where I can report this (30-39, female, Law, Arts and Humanities, assistant/associate prof, temporary, 6-10 years).

We also examined the variation in researchers with permanent and temporary work contracts’ types of responses. The results indicate that researchers in permanent positions confront the culprits more often (26%) than those in temporary positions (14%). Such confrontations often involved students or other co-authors, as in the following example:

[I] talked to the persons involved, co-authors, and reported [this misconduct] to a faculty representative specialized in misconduct (60-69, male, Medical and Life Sciences, professor, permanent, 16+ years).

Researchers in permanent positions also informed their superiors more often (26%) than those in temporary positions (14%). Similarly, researchers in temporary positions ‘did nothing’ more often (23%) than their colleagues in permanent positions (15%). These researchers also talked to colleagues more often (24%) than those in permanent positions (11%). In the qualitative responses, these statements were usually not explained or narrated, but emerged in the form of “I did nothing” or “I discussed it with my colleagues”. However, there were noteworthy exceptions:

Nothing, because the present head of department (new last author [of] this paper) tries to eliminate me and I am completely dependent [on] him (female, 60-69, Medical/Life Sciences, assistant/associate prof, temporary, 16+ years).

Nothing, due to the principal researcher’s wish (30-39, male, Medical/Life Sciences, temporary, assistant/associate prof, 0-5 years).

Here too, the dependency on research and career resources that staff members in more permanent positions control seems crucial in terms of the use of power relations.

Finally, we examined the variation in researchers with temporary and those with permanent positions’ perception of the outcomes of reporting. Our data suggest that a larger percentage of researchers with permanent positions report a constructive change, as shown in the following:

[I] asked the editor of the journal to withdraw the paper; the thesis itself did not suffer, as the citation and reference were included in another chapter in the same thesis (60-69, male, Medical and Life Sciences, professor, permanent, 16+ years).

This person got fired (30-39, female, Social and Behavioural Sciences, associate/assistant professor, temporary, 6-10 years).

Only researchers in permanent positions reported negative outcomes (7%), such as “the relationship with the author involved is somewhat troubled” (60–69, female, Medical and Life Sciences, professor, permanent, 11–15 years). Consequently, while a fear of reporting’s potential negative consequences is often presented as a reason for not reporting a case, there are hardly any known or acknowledged consequences at all in practice. This refers specifically to researchers with temporary contracts.

Finally, researchers in temporary positions reported ‘no change’ to a greater extent (55%) than those in permanent positions (38%). These responses were usually not narrated, but expressed in the form of “no change” or “Not much—but at least more of my earlier work is cited” (30–39, male, Natural Sciences, assistant/associate professor, temporary, 6–10 years).

In our sample, therefore, researchers in permanent positions reported misconduct more often than those in temporary positions, and their reporting is more likely to have a constructive result. This indicates that, in academia, the type of work appointment may have an effect on the reporting practices and their outcomes. Possible reasons for the difference between the two groups are that researchers in temporary positions feel they have more to lose by reporting and are less interested in it, because they identify less with the work organisation.

#### Gender

The third variable we explore concerns gender differences in the reporting of alleged misconduct and the consequences of this. This exploration was based on the literature’s assertion that men are more likely to report misconduct and to perceive the consequences as more constructive than women. However, contrary to our expectation and as we will show, few of the differences are related to gender.

There was little difference between men and women regarding the reporting of alleged research misconduct. 51% (N = 50) of the men and 45% (N = 40) of the women claimed to have reported a witnessed case of misbehaviour.

Neither does there seem to be substantial gender differences regarding reactions to misconduct. With regard to the most commonly reported forms of acting upon cases of alleged misconduct, 24% of women and 20% of men reported having confronted the culprits. The same is true of ‘talking to colleagues’, which 19% of women and 14% of men did, and of 'informing supervisor’, which 20% of women and 22% of men did. ‘No response’ was selected by 16% of the women and 18% of the men.

Finally, our data show that there are no substantial differences between men (27%) and women’s (26%) perceptions of reporting having constructive consequences. Women report a slightly higher number of negative consequences (6%) than men (3%), but the relative difference is small and the absolute number is so low that we cannot draw any clear conclusions from this result.

In the qualitative responses, there were also no noteworthy differences between men and women regarding how the instances of the reporting of misconduct and reactions to this were articulated and made sense of. None of the responses used wording related to gender. In terms of our data, gender does not seem to be distinctively related to researchers’ reporting of misconduct, or to the outcomes of their reporting.

### Type of Misconduct Reported

The last variable we analysed concerns the type of misconduct witnessed. Although not directly related to power structures, this variable probably influences reporting behaviour, since more clear-cut types of research misconduct may be more readily reported than more nuanced forms of misconduct (Near and Miceli 1985; Mesmer-Magnus and Viswesvaran 2005). We distinguish between fabrication, falsification and plagiarism (FFP) as clear-cut forms of misconduct, and the rest (QRP) as more nuanced forms of misconduct (see “Appendix”).

Table 2 shows that plagiarism was the most commonly reported type of misconduct, followed by authorship issues, fabrication, cherry picking, falsification, text recycling, and data manipulation. Taking relative rather than absolute numbers into consideration, we conclude that the more contentious forms of misconduct, such as authorship and cherry picking, have a lower reporting ratio than the more clear-cut forms, such as plagiarism, falsification and fabrication.

**Table 2:** Types of misconduct reported

Was it reported?	Yes	No	Don't know	NA
Plagiarism (N = 60)	38 (63%)	14 (23%)	5 (8%)	3 (5%)
Authorship (N = 49)	16 (33%)	31 (63%)	0 (0%)	2 (4%)
Cherry picking (N = 28)	9 (32%)	18 (64%)	1 (4%)	0 (0%)
Falsification (N = 14)	6 (43%)	7 (50%)	0 (0%)	1 (7%)
Fabrication (N = 13)	11 (85%)	2 (15%)	0 (0%)	0 (0%)
Text recycling (N = 7)	5 (71%)	2 (29%)	0 (0%)	0 (0%)
Data manipulation (N = 5)	3 (40%)	2 (40%)	0 (0%)	0 (0%)
Don't understand (N = 5)	2 (40%)	3 (60%)	0 (0%)	0 (0%)
Do not wish to answer (N = 1)	1 (100%)	0 (0%)	0 (0%)	0 (0%)

We only include the subset of the total sample in the tables, only including forms of misconduct where N > 5

We also examined the perceived outcomes of reporting with regard to the different forms of misconduct. Table 3 shows that plagiarism has the highest number of constructive consequences related to reporting, followed by cherry picking and falsification. In terms of no change, authorship has the highest number, followed by plagiarism and cherry picking.

**Table 3:** Perceived consequences of reporting

Did anything change?	Constructive consequences	Negative consequences	No change	Don't know	NA
Plagiarism (N = 60)	20 (33%)	3 (5%)	19 (32%)	10 (17%)	8 (13%)
Cherry picking (N = 28)	8 (29%)	0 (0%)	14 (50%)	0 (0%)	6 (21%)
Falsification (N = 14)	7 (50%)	0 (0%)	6 (43%)	0 (0%)	1 (7%)
Fabrication (N = 13)	6 (46%)	3 (23%)	4 (31%)	0 (0%)	0 (0%)
Authorship (N = 49)	4 (8%)	1 (2%)	35 (71%)	2 (4%)	7 (14%)
Text recycling (N = 7)	3 (43%)	1 (14%)	2 (29%)	0 (0%)	1 (14%)
Data Manipulation (N = 5)	2 (40%)	0 (0%)	3 (60%)	0 (0%)	0 (0%)
Don't understand (N = 5)	2%	0%	40%	0%	40%
Do not wish to answer (N = 1)	100%	0%	0%	0%	0%

These numbers also reflect the contours of the difference between the clear-cut forms of misconduct and the more contentious forms. Likewise, as we noted earlier, there is a generally remarkably low chance of experiencing a positive, constructive consequence of reporting, with a much higher rate of respondents mentioning perceiving no changes at all.

Overall, therefore, we found that clear-cut cases of misbehaviour are reported more often than nuanced cases. Since nuanced cases are difficult to assess normatively, which could make a (formal) misconduct case a precarious endeavour, this could explain the difference. Likewise, given the difficulty of justifying such accusations, researchers may feel it is a too uncertain undertaking, risking long, intensive procedures and potential repercussions. Several respondents did indeed indicate as much in their answers:

No... since it's not actually forbidden, but still [an] unethical research practice (cherry picking, referring to “[w]ild and unjustified analyses until there is a significant result”; 30-39, female, Social Sciences, PhD student, temporary, 0-5 years).

I reported to the direct supervisor, but was sure I could not go beyond that, as that would directly have impeded my own relation with my own supervisor (I am a PhD student). Since this is an instance of [an] incomplete assessment of [an] error on results, this does not seem to classify as direct misconduct (20-29, male, natural sciences, PhD student, temporary, 0-5 years).

## Discussion

Our survey results suggest that demographic differences affect the likelihood that alleged misconduct cases are acted upon, i.e. reported and dealt with constructively. Firstly, the analysis suggests that younger researchers, researchers with temporary appointments and those in lower academic positions are less likely to report misconduct, compared to their more senior and permanently appointed colleagues. Secondly, contested forms of misconduct (e.g. authorship, cherry picking of data) seem to be reported less than more clear-cut instances of misconduct (e.g. plagiarism, text recycling and the falsification of data).

These trends, which emerge quantitatively, can be meaningfully interpreted in combination with the qualitative data. In the respondents’ answers to the open-ended survey questions, they frequently attribute their decisions regarding whether and how to report to hierarchy or power issues. Some respondents do this very explicitly by referring to their previous superior, or to their current hierarchical relationship with their supervisor. Others hint more implicitly at hierarchy and power issues, implying power imbalances in the sense of resource dependence.

Based on the numerical data and our respondents’ interpretation of these and the context, we can refine and translate the general claims made in the whistleblowing literature into specific propositions on reporting alleged research misbehaviour at academic institutes. Owing to the sample size limitations and the high diversity among participating research institutes, our results might be insufficient to draw definitive, statistically relevant conclusions, but we do believe that they provide strong indications that future studies could verify. We will formulate the propositions relating to the variables studied separately: academic seniority, the temporality of work contracts, gender, and the type of misconduct witnessed.

### Academic Seniority

The first set of statements involves academic seniority and age. Overall, the results suggest that seniority in research constitutes a valuable resource in respect of reporting alleged research misconduct. This arguably manifests itself through access to important resources during the reporting process. Our respondents maintained that these resources include, for example, the knowledge to identify such misconduct and the most suitable reporting channel, the social capital and power position to act on misconduct (e.g. due to the low perceived career risk), as well as the ability to follow up on cases in order to secure a constructive outcome. Junior researchers may not possess the same knowledge and social capital, and fear harming their academic careers. These exploratory findings suggest the following propositions for further hypothesis-testing research:

Proposition 1: Researchers in junior academic positions and younger researchers are less likely to report instances of alleged research misconduct compared to more senior and older researchers.

Proposition 2: Reporting by researchers in junior positions or younger researchers is less likely to lead to constructive consequences compared to that of more senior and older researchers.

### Work Appointment

The second set of statements involves the temporality of work appointments. It is clear that for researchers and all other forms of employees, temporary contracts constitute an element of power imbalance. Temporary employment essentially entails that these employees are excluded from tenured work agreements and therefore face the risk of their work contracts not being renewed. It is reasonable to believe this, and our results also provide initial evidence of such a lack of power and social capital manifesting itself in these researchers’ reporting behaviour. For example, their fear of negative personal consequence in the aftermath of reporting alleged misconduct, which respondents with temporary contracts expressed more often than others, is a manifestation of their lack of power. This leads us to the second set of propositions:

Proposition 3: Researchers with temporary contracts are less likely to report instances of alleged research misconduct than those with permanent contracts.

Proposition 4: Reporting by researchers with temporary contracts is less likely to lead to constructive consequences than reporting by those with permanent contracts.

### Gender

The third set of statements involves gender. Somewhat surprisingly, we did not find any strong indications that gender differences play a role in reporting alleged research misconduct. However, we believe the theoretical underpinnings of gender as a central dimension of power imbalances in organisations and in academia more specifically (Aagaard 2016; Grilli and Allesina 2017; Treviño et al. 2017) are strong enough to warrant further studies. Contrary to our findings, we thus propose the following propositions:

Proposition 5: Female witnesses of alleged research misconduct are less likely to report such instances than their male colleagues.

Proposition 6: Reporting by female researchers is less likely to lead to constructive consequences than reporting by male researchers.

### Type of Misconduct Reported

The fourth and final statement category involves the characteristics of the alleged misconduct. Although not a distinct power dimension, it does shed light on the likelihood of researchers’ perception that their complaint will be regarded seriously, acted upon and lead to constructive consequences. Given that clear cases of misconduct are easier to identify and their reporting easier to justify (Miceli and Near 2005), we propose the following final statement:

Proposition 7: Researchers are more likely to report clear-cut instances of alleged research misconduct than more nuanced or ‘grey areas’ of misbehaviour.

The lack of reporting of ‘grey’ forms of misconduct is due to the crucial negative effect of such forms of misconduct being potentially continued. In other words, not only are such forms of misconduct per definition difficult to assess normatively, they are also likely to be more unspoken and implied in research. This involves the risk of such practices becoming embedded and institutionalised rather than openly discussed and reflected upon. Indeed, institutional or national integrity committees’ processing of allegations of misconduct has often led to the codification of research practices (Horbach et al. 2018). Consequently, if cases of specific types of alleged misconduct are not reported and integrity committees cannot assess them subsequently, they may not be classified as either proper or improper research practices. The assessment of such research practices is hence in need of further research.

## Conclusion and Recommendations

In this study, we have analysed reporting as one of the social control mechanisms flagging misbehaviour in science. In particular, we have studied the actors who are most likely to report alleged misconduct, how they report, and the consequences of reporting. We found differences in the rate of reporting and the consequences thereof, depending on the demographic characteristics of the person witnessing the case.

These insights contribute to the literature on research misconduct in two ways. Firstly, to our knowledge, we provide the first systematic insights into researchers’ reasons and explanations for reporting, or not reporting, witnessed misconduct. We find indications that younger researchers, researchers with temporary appointments and those in lower academic positions are less likely to act and report than their senior and permanently appointed colleagues. The crucial hurdles for not reporting are these researchers’ concerns that this may harm their career and their expectation of not being taken seriously, both of which are rooted in power relations and hierarchical differences leading to resource dependence.

We also find that contested forms of misconduct (e.g. authorship, cherry picking of data and fabrication of data) are less likely to be reported than more clear-cut instances of misconduct (e.g. plagiarism, text recycling and falsification of data). The respondents mention that minor misbehaviour is not considered worth reporting, or express doubts about the effectiveness of reporting a case when the witnessed behaviour does not explicitly transgress norms, such as with many of the QRPs. Concern about reporting’s negative consequences, such as career opportunities or organisational reputations being harmed, is always taken into considerations.

Secondly, we have theorised the relationship between power differences and researchers’ willingness to report—in particular the role of seniority, work appointments and gender. We have derived a list of seven propositions that we believe warrant testing and refinement in future studies using a larger sample to help with further theory building about power differences and research misconduct. More specifically, by focusing on such structural power dimensions, we provide a different perspective than most prior studies of scientific misconduct, which have mainly focused on the negative consequences for the individual wrongdoer and his/her colleagues. We thus open up a broader organisational understanding of the mechanisms that impact researchers’ ability and willingness to successfully report misconduct.

Based on our study, we argue that establishing adequate reporting procedures is a prime requirement to empower less powerful members of the research community to report scientific misbehaviour. This may also specifically strengthen one of science’s most important social control mechanisms in which direct colleagues check each other’s work. Following Lukes’s and Bachrach and Baratz’s conception of power in the form of agenda setting (Lukes 2005; Bachrach and Baratz 1962), reporting procedures are a prime way of making latent and covert interests visible, thereby demanding decisions from those in power.

Our findings may have several implications for policy. We argue that policy interventions, such as research integrity courses for junior researchers, the articulation of research integrity codes, or integrity boards have to consider the power imbalances in research organisations. Our results suggest a need for improved reporting procedures. Specifically, such procedures should take the position of an organisation’s less powerful members, such as junior researchers and people with temporary work appointments, into account and facilitate their reporting. This requires procedures that effectively address issues of power imbalance and the fear of not being taken seriously. The implementation of such procedures could help target a culture of complacency and cynicism that normalises questionable research practices. In addition, it may contribute to a sense of organisational responsibility that should ultimately foster a climate of research integrity.

### Electronic supplementary material

Below is the link to the electronic supplementary material.

Supplementary file1 (PDF 701 kb)

Supplementary file2 (XLSX 73 kb)

## Appendix: Overview of coding and illustrative examples

**Table:**

Question category	Analytic (second-order) categories	Responses (first-order codes)	Examples
Form of misconduct (N = 202)	FFP	Plagiarism (N = 60, 30%)	“[…] a professor was using my unpublished material in an article without asking and without citing me properly.” (40–49, Female, Social and behavioral sciences, Associate professor, Permanent, 6–10 years)
		Falsification (N = 14, 7%)	“Most negative controls in an [PhD study] experiment were copy/paste of the same data.” (40–49, Male, Medical and life sciences, Associate professor, Temporary, 16 + years)
		Fabrication (N = 12, 6%)	“Making up data” (40–49, Female, Medical and life sciences, Associate professor, Permanent, 16 + years)
	QRP	Authorship (N = 47, 23%)	“Another supervisor claimed co-authorship without contributed to writing” (60–69, Male, Social and behavioral sciences, Professor, Permanent, 16 + years)
		Cherry picking (N = 29, 14%)	“Cherry picking of data based on pre-decided outcome” (40–49, Male, Social and behavioral sciences, Professor, Permanent, 11–15 years); “‘Fishing’ until a statistically significant finding was found” (30–39, Female, Social and behavioral sciences, Associate professor, Permanent, 6–10 years)
		Text recycling (N = 7, 3%)	“Several cases of self-plagiarism, but of a fairly innocent kind (paragraphs about methods)” (40–49, Male, Natural sciences, Professor, Permanent, 16 + years)
		Withholding data (N = 5, 2%)	“A project initiated by myself and my co-worker was 'stolen' aggressively by project collaborators after a while. Microscopic scientific photographs made by us were published without our consent. Access to certain resources were blocked.” (50–59, Female, Natural sciences, Professor, Permanent, 16 + years)
		Lack of approval (N = 5, 2%)	“Researcher started measurements (not invasive but taking pictures of the skin) but did not have all informed consent forms yet (though he would do this after measurements)” (30–39, Female, Medical and life sciences, Postdoc, Permanent, 6–10 years)
		False reporting of number of articles (N = 3, 1%)	“[…] putting the same articles as output on several, different grants. Even articles that I published as a post-doc in a different university ended up at this grant-evaluation, whereas I hadn't written them during my time at that university. And without my knowledge, by the way.” (30–39, Female, Social and behavioral sciences, Associate professor, Temporary, 11–15 years)
		Lack of citations (N = 3, 1%)	“A colleague in my department published a book without my 7 contributions. He praised the contributions before we became involved in a severe conflict.” (50–59, Female, Law arts and hum, associate professor, Permanent, 16 + years)
		Mistreatment of research subjects (N = 3, 1%)	“Mistreatment of research primates, resulting in severe psychological suffering of animals, physical illness & risks, including animals who died from it” (20–29, Female, Natural sciences, PhD student, Temporary, 6–10 years)
		Peer-review fraud (N = 2, 1%)	“Block paper as referee and then publish a paper oneself on the topic” (60–69, Male, Natural sciences, Scientific director, Permanent, 16 + years)
		Pressure by senior or study contractor (N = 2, 1%)	“I was working with a more senior professor to promote findings of some research through a prestigious impact/knowledge mobilisation event […].[…] he called me to a teleconference with the sponsor of the event and they both put a lot of pressure on me to allow the tool to be presented as effective.” (40–49, Female, Medical and life sciences, Associate professor, Permanent, 16 + years)
		Redundant publication (N = 2, 1%)	“Publishing two very similar articles under different titles” (50–59, Male, [field unknown], Professor, Permanent, 16 + years)
	No answer (N = 3, 1%)		
How did you become aware of the misconduct (N = 194)	First-hand experience (N = 111, 57%)	Direct involvement or witness (N = 37, 19%)	“Discovered it myself in previous job as a Research Associate” (40–49, Social and behavioral sciences, Female, PhD student, Temporary, 16 + years)
		Reading text (N = 23, 12%)	“Read an early draft of an article” (40–49, Male, Social and behavioral sciences, Professor, Permanent, 11–15 years)
		Collaboration (N = 16, 8%)	“By working with the researcher in question” (20–29, Female, Social and behavioral sciences, PhD student, Temporary, 0–5 years)
		As an editor (N = 14, 7%)	“Being editor in chief of some journals I see many cases of misconduct every year. Usually through readers of the journal that recognize e.g. plagiarism or redundant publication” (60 +, Male, Natural sciences, Professor, Permanent, 16 + years)
		As a reviewer (N = 11, 6%)	“While reviewing a submission for a well-known journal” (30–39, Female, Law, arts and hum, Associate professor, Permanent, 6–10 years)
		Hearing a presentation (N 5, 3%)	“Presentation of a colleague within the department before Ph.D. defense.” (20–29, Male, Natural sciences, Ph.D. student, Temporary, 0–5 years)
	Second-hand experience (N = 75, 39%)	From colleague (N = 45, 23%)	“The person who discovered it (the person who misconducted was her team member) shared it with me and other colleagues” (20–29, Female, Medical and life sciences, Ph.D. student, Temporary, 0–5 years)
		From culprit (N = 10, 5%)	“Researcher happily telling me by email he had submitted a paper which I and he knew to contain major flaws” (40–49, Male, Natural sciences, Professor, Permanent, 16 + years)
		From peers (N = 6, 3%)	“Emails from colleagues abroad” (50–59, Male, Social and behaviour sciences, Associate professor, Permanent, 16 + years)
		From student (N = 6, 3%)	“I was consulted by a Ph.D. student who disagreed with his supervisor on the interpretation and presentation of results” (40–49, Male, Medial and life sciences, Associate professor, Permanent, 16 + years)
	No answer (N = 6, 3%)		
How did you react (N = 205)	Specific reaction (N = 168, 77%)	Talk to superiors (N = 73, 33%)	“Order investigation to see if plagiarism also had been committed during the period that this colleague worked in our institution” (50–59, Male, Law, arts and hum, Professor, Permanent, 16 +)
		Confront culprits (N = 44, 20%)	“I told her it was not OK. When things escalated, I also told representative from the financier of the project and I also told the manager of the faculty.” (30–39, Female, Social and behavioral sciences, Professor, Permanent, 11–15 years)
		Discuss with colleagues (N = 21, 10%)	“Talked to two colleagues, one of whom had unsuccessfully addressed a similar issue with the research team in question earlier” (40–49, Male, Social and behavioral sciences, Professor, Permanent, 11–15)
		Conduct own investigation (N = 8, 4%)	“Check all the raw data in the daily logbooks” (50–59, Male, Medical and life sciences, Associate professor, Permanent, 16 + years) “Investigated contributions to volumes co-edited by me” (60–69, Female, Language, information and communication, Professor, Permanent, 16 + years)
		Change own practice (N = 7, 3%)	“We plead guilty and resynthesised most of the compounds presented in the paper” (40–49, Male, Natural sciences, Associate professor, Permanent, 16 + years)
		Withdraw from collaboration (N = 6, 3%)	“I gave up the article, because I didn't want to be involved in these kinds of practices: it wasn't worth it.” (20–29, Female, Medical and life sciences, Ph.D. student, Temporary, 0–5)
		Exerting voice (N = 6, 3%)	“Drew people's attention to it” (60 +, Female, Natural sciences, Professor, Temporary, 16 + years)
	No reaction (N = 37, 17%)		“I first demonstrated that it was not deserved, but because of university pressure, I gave up and allowed it” (60 +, Male, Social and behavioral sciences, Professor, Permanent, 16 + years)
	No answer (N = 14, 6%)		
Was the instance reported? (N = 194)		Reporting (N = 89, 46%)	“Yes, but there was no ‘fingerprint’ proof, so that didn't help.” (60 +, Male, Natural sciences, Professor, Permanent, 16 + years)
		No reporting (N = 90, 46%)	“No. My department manager never takes any action on any problem.” (40–49, Female, Social and behavioral sciences, PhD student, Temporary, 0–5 years)
Did anything change? (N = 193)		No change (N = 87, 45%)	“No, this was a 'mistake' by a single individual, probably under time pressure. We (his colleagues) usually discuss all results openly, and we should have noticed this before.” (40–49, Male, Medical and life sciences, Associate professor, Temporary, 16 + years)
		Constructive chance (N = 52, 27%)	“Yes, the whole field changed its way of conducting research and dealing with data” (30–39, Female, Social and behavioural sciences, Associate professor, Temporary, 6–10 years)
		Negative change (N = 8, 4%)	“It caused misplaced insecurity in the physics community with regard to integrity matters.” (50–59, Male, Natural sciences, Professor, [position DNR], 16 + years) “I left academia because I've had enough. This situation has been going on for years, and I'm not able to change it” (30–39, Male, Social and behavioural sciences, left academia, 6–10 years)
	Don’t know (N = 15, 8%)		
	No answer (N = 31, 16%)		
