# Supplementary material for: On the Willingness to Report and the Consequences of Reporting Research Misconduct: The Role of Power Relations
Source: Sci Eng Ethics. 2020 Feb 26;26(3):1595–623. doi: 10.1007/s11948-020-00202-8 (PMC7286863; doi:10.1007/s11948-020-00202-8)
Supplement: Supplementary file 1 — Supplementary file1 (PDF 701 kb) [file 11948_2020_202_MOESM1_ESM.pdf]

Printeger survey

In this survey, we will gather data on organisational policies on misconduct and integrity; whistleblowing mechanisms and attitudes; work environment features; perceptions of tensions and risks associated with misconduct and compromise of scientific quality; perceptions of integrity measures; and prevalence of misconduct. We will also ask some background questions.

The information gathered in this survey will be secured so that it is accessible only to the PRINTEGER research team in Oslo. Even if it might in some cases be theoretically possible to identify individuals by connecting background variables, this will not be done in the analysis, nor in the reporting of the data. We will not present comparative data naming the organisations included in the survey. Data will be securely stored at a password-encrypted PC for five years after the project is completed: then the data will be deleted.

In filling out the survey you should not reveal any information about individual persons. If you would like to report cases of misconduct, we encourage you to use the channels that are available in your organisation/country.

The survey has been approved by the relevant research ethics or data protection authorities in the PRINTEGER partner countries.

No data will be saved until the form is submitted. Because your answers will not be traced to your name and your name is not registered with any electronic identity, it will not be possible for us to remove your individual answers once they have been submitted in the web form.

If you have questions about the survey or about the project, please do not hesitate to contact the PRINTEGER partner in your country http://printeger.eu/consortium/, the partner responsible for the survey (Eric.Breit@afi.hioa.no) or the project coordinator (H.Zwart@science.ru.nl).

The following information about hidden identity is standard information from the survey provider Questback Essentials:

1) Consent to participate

- ○ I consent to participate in the survey
- ○ I do not consent to participate in the survey.

ORGANISATIONAL POLICIES

In this section we are interested in your research institution's policies for raising awareness of research misconduct and integrity.

2) Does your research institution have a written policy about research misconduct and integrity at the following levels?

	Yes	No	I don't know
My department	○	○	○
My faculty	○	○	○
My university	○	○	○

3) On a scale from 1 to 10 with 1 being "very unsure" and 10 being "very sure":

	1	2	3	4	5	6	7	8	9	10
How confident are you in your understanding of research misconduct?	○	○	○	○	○	○	○	○	○	○

4) How are new employees introduced to research integrity in your department? (Several answers possible)

- ☐ No particular introduction
- ☐ Ethics course
- ☐ Oral introduction to ethical guidelines
- ☐ Written information about ethical guidelines
- ☐ I don't know

5) How consistently do managers in your department communicate high expectations for research integrity?

- Not at all
- Somewhat
- Moderately
- Very
- Completely
- No basis for Judging

Here we are interested in the mechanisms for and attitudes towards whistleblowing when it comes to research misconduct.

6) To what degree would you feel responsible to report internally or externally the suspected misconduct if you witnessed any of the following

	Not at all	To a small degree	To a certain degree	To a large degree
Fabrication of data				
Plagiarism				
Falsification of data (i.e. manipulating research materials without scientific justification)				
Selective dropping of data from “outlier” cases without transparent explanation				
Trying out a variety of different methods of analysis until one is found that yields a result that is statistically significant				
Withholding data from the research community				
Falsification of bio-sketch, resume or personal reference statements				
Non-disclosure of a conflict of interest				
Pressure from a study sponsor or contractor to engage in unethical research conduct or skewed presentation of research				

7) Do you agree with the following statements about whistleblowing?

	Not at all	To a small degree	To a certain degree	To a large degree
I know the appropriate routines for whistleblowing in the event of witnessing misconduct	○	○	○	○
I feel confident that I would be protected as a whistleblower	○	○	○	○
I feel confident that the faculty (or other relevant bodies in the university) would take seriously the whistleblowing and act accordingly	○	○	○	○

8) If you witnessed or heard about suspected misconduct, with whom would you discuss this? (Several answers possible)

- ☐ Persons involved in the misconduct
- ☐ Department colleagues generally
- ☐ Research managers
- ☐ Department manager
- ☐ Research ethics committee staff
- ☐ Ombudsman
- ☐ Union representative
- ☐ Media
- ☐ Nobody
- ☐ Other, please specify [   ]

WORK ENVIRONMENT

In this section we are interested in how you perceive aspects of your work environment that may be relevant to research misconduct and integrity.

9) In your immediate work environment, how would you rate the following?

	Very low	Low	Medium/average	High	Very high	I don't know
Availability of academic positions	○	○	○	○	○	○
Difficulty in obtaining tenure	○	○	○	○	○	○
Pressure on researchers to obtain external funding	○	○	○	○	○	○
Pressure on researchers to publish	○	○	○	○	○	○
Severity of penalties for research misconduct	○	○	○	○	○	○
Chances of getting caught for research misconduct if it occurs	○	○	○	○	○	○
Researchers’ understanding of rules and procedures related to research misconduct	○	○	○	○	○	○
Management understanding of rules and procedures related to research misconduct	○	○	○	○	○	○
My own understanding of rules and procedures related to research misconduct	○	○	○	○	○	○
Researchers’ support of rules and procedures related to research misconduct	○	○	○	○	○	○
Management support of rules and procedures related to research misconduct	○	○	○	○	○	○
My own support of rules and procedures related to research misconduct	○	○	○	○	○	○

10) In your department are there direct economic incentives (other than career advancement) for you individually related to

	No	Yes	I don't know	I do not whish to answer
Acquisition of research funding	○	○	○	○
Publication of scientific articles	○	○	○	○
Other	○	○	○	○

11) To what degree do you agree/disagree with the following statements about the work environment in your department?

	Strongly disagree	Disagree	Neither disagree nor agree	Agree	Strongly agree
The work culture at my department is oriented more towards individual performances than towards collective performances	○	○	○	○	○
I conduct most of my research alone rather than in collaboration with colleagues	○	○	○	○	○
It is easy for my colleagues and managers to monitor and assess my research	○	○	○	○	○
In my department there is a strong hierarchy between senior and junior researchers	○	○	○	○	○
The culture of my department is supportive of openly resolving ethical concerns or research errors	○	○	○	○	○
There is a shared understanding of what is good research conduct in my department	○	○	○	○	○
My department managers' focus on research integrity is strong	○	○	○	○	○
I frequently assess my own performance (for instance my H index, Research Gate score etc.) compared to that of my peers	○	○	○	○	○
I have an active role in academic networks, associations or societies outside my own department	○	○	○	○	○
In my department there is a culture for open discussion about research misconduct and integrity	○	○	○	○	○
In my department there is a high level of pressure to commercialize results or outcomes of research	○	○	○	○	○
In my department there is a high level of time and workload pressure regarding research activities	○	○	○	○	○

12) In your department, how guarded are people in their communications with each other out of concern that someone else will "steal" their ideas?

- ○ Not guarded at all
- ○ Somewhat guarded
- ○ Guarded
- ○ Very Guarded

TENSIONS AND RISK

Here we are interested in what you perceive as risks related to research misconduct.

13) If you work in projects or take part in academic networks with scientists from other institutions, countries or fields, do you experience conflicting standards regarding proper research conduct?

- Never
- Seldom
- Sometimes
- Often

14) Do you experience a tension between loyalty to academic values of rigorous research and loyalty to

	Never	Seldom	Sometimes	Often
Funding bodies				
The department management				
Co-workers				
The subjects of research				

15) In my faculty, I perceive research misconduct to be a minor problem

- Strongly disagree
- Slightly disagree
- Neither agree nor disagree
- Agree slightly
- Agree strongly

16) In my field of research, I perceive research misconduct to be a minor problem

- Strongly disagree
- Slightly disagree
- Neither agree nor disagree
- Agree slightly
- Agree strongly

17) In research in general, I perceive research misconduct to be a minor problem

- Strongly disagree
- Slightly disagree
- Neither agree nor disagree
- Agree slightly
- Agree strongly

18) How do you rate the risk that you might be personally implicated in research misconduct or questionable research practices?

- Impossible
- Very unlikely
- Unlikely
- Neither likely, nor unlikely
- Likely
- Very likely

RESEARCH QUALITY

Compromised research quality may in some situations be a question of research integrity and we are therefore also interested in factors that may affect quality.

19) Have any of the funders of research projects you have been involved in ever unduly interfered in your work?

- No
- Yes
- Do not wish to answer

20) You answered "yes" to the prior question that any of the funders of research projects you have been involved had ever unduly interfered in your work. Can you please shortly elaborate?

21) Do you believe that the quality of your research varies depending on the funding source?

- No
- Yes
- I don't know
- I do not wish to answer

22) Does the quality of your research

	Yes	No	Not sure
Suffer due to strict time constraints?			
Suffer due to insufficient available data?			
Suffer due to other reasons			

23) You answered "Yes" on the prior question "Does the quality of your research suffer due to other reasons". Could you please spesify?

24) Do you believe that you have a better chance of getting your research published if you draw stricter conclusions and avoid mentioning uncertainties in the abstract/conclusion of your study (but rather in a limitations section)?

- Yes
- No
- I don't know

25) Do you attempt to publish findings also when they are negative or inconclusive in respect to a specific research question?

Always | Sometimes | Rarely | Never | Not relevant

INTEGRITY MEASURES

Here we are interested in how you perceive potential integrity measures.

26) In order to strengthen research integrity and/or reduce the risk of misconduct, to what degree do you agree that the following measures are useful

	Strongly disagree	Disagree	Neither disagree nor agree	Agree	Strongly agree
Increased monitoring internally (by managers)	○	○	○	○	○
Increased monitoring internally (by peers)	○	○	○	○	○
Increased monitoring externally (for instance by research funding organisations)	○	○	○	○	○
Change of performance criteria and rewards	○	○	○	○	○
Ethical reflection groups and open dialogue	○	○	○	○	○
Managerial emphasis and attention to research integrity	○	○	○	○	○
Increased severity of sanctions	○	○	○	○	○
Information on ethical guidelines	○	○	○	○	○
Online training tools	○	○	○	○	○
Conventional training and education in research ethics	○	○	○	○	○

PREVALENCE OF RESEARCH MISCONDUCT

Here we are interested in your personal experience with research misconduct.

27) Have you known about or justifiably suspected that any of the colleagues in your faculty during the last 12 months has

	Yes	No	Uncertain/do not know	Do not want to answer	Not applicable
Fabricated data	○	○	○	○	○
Plagiarized data	○	○	○	○	○
Falsified data	○	○	○	○	○
Selectively dropped data from "outlier" cases without transparent explanation	○	○	○	○	○
Tried out a variety of different methods of analysis until one is found that yields a result that is statistically significant	○	○	○	○	○
Deliberately withheld data from the research community to gain personal or institutional advantage	○	○	○	○	○
Falsified biosketch, resume, reference list	○	○	○	○	○
Not disclosed a conflict of interest	○	○	○	○	○
Claimed undeserved authorship	○	○	○	○	○
Denied authorship to contributors	○	○	○	○	○
Been pressured by a study sponsor or contractor to engage in unethical research conduct or skewed presentation of research	○	○	○	○	○

28) Have you during the last 12 months been exposed to unethical pressure concerning

	Yes	No	Uncertain/do not know	Do not want to answer	Not applicable
Ordering/inclusion of authors	○	○	○	○	○
Design/method	○	○	○	○	○
Analysis of data	○	○	○	○	○
Presentation of results	○	○	○	○	○

29) If you answered 'yes' to having been exposed to unethical pressure; please indicate the sources of the pressure (several answers possible):

- [ ] The commissioner/funder of the research
- [ ] Stakeholders with interest in the research
- [ ] Colleagues in my faculty
- [ ] A manager in my faculty
- [ ] Colleagues outside my faculty
- [ ] Colleagues or managers at a former employer
- [ ] Not appplicable
- [ ] Other, please specify

30) If you have first-hand knowledge of specific situations of research misconduct please select one situation and answer the following questions. Please do not include any identifying information.

- ○ Yes, I do have a first-hand knowledge of a case
- ○ No, I do not have first-hand knowledge of a case
- ○ I do not wish to answer

31) You have a first-hand knowledge of case of research misconduct. Can you please elaborate on the following questions

How did you first learn about the instance of research misconduct?	
Please describe the specific instance of research misconduct	
What did you do when you became aware of it?	
Whom (titles only) did you talk with about the research misconduct? How did you feel during this experience?	
Were you able to talk with the individual(s) who were involved about it? Please describe your interaction.	
Was the instance reported? If so, to whom and by whom? Whether the decision was to report or not, how was the decision made? What were the factors underlying the decision to make the report?	
What was the outcome? How did you feel about the way it was handled?	
Did you think anything changed as a result?	
Is there anything you would have done differently?	

BACKGROUND QUESTIONS

Here we would like to gain some more information about background variables that might be relevant to explain experiences and that will help us design well-targeted recommendations for effective integrity measures.

32) How many academic (peer-reviewed) publications (including co-authored papers) have you authored over the last year?

○ 0 ○ 1-2 ○ 3-5 ○ 6-9 ○ 10 or more

33) Do you belong to any association for researchers?

- Yes
- No
- Not applicable
- Do not want to answer

34) How many lectures, workshops, or conferences on research ethics have you attended (or held) in the past year?

- 0
- 1-5
- 6-10
- 11 or more

35) How satisfied are you with your current work situation (i.e. your well-being at work)?

- Very satisfied
- Quite satisfied
- Neither satisfied nor dissatisfied
- Quite unsatisfied
- Not satisfied at all

36) To what extent do you identify with the professional culture and values of your department?

- To a large extent
- To a certain extent
- To a small extent
- Not at all
- Don’t have an opinion
- Don’t know

37) Age

- 20-29
- 30-39
- 40-49
- 50-59
- 60-69
- 70 and older

38) Gender

- Male
- Female
- Other

39) Employed at

- University of Bonn
- University of Bristol
- Free University of Brussels
- University of Leiden
- Radboud University
- University of Tartu
- University of Trento
- Oslo and Akershus University College of Applied Sciences
- Other/Do not wish to answer

40) Highest academic degree

- Doctoral degree
- Master’s degree
- Bachelor’s degree
- Other degree

41) Academic field (of your highest degree)

- Engineering sciences
- Language, information and communication
- Law, arts and humanities
- Medical and life sciences
- Natural sciences
- Social and behavioral sciences

42) Do you currently have a manager or leadership role?

- No
- Yes

43) Current academic position

- Professor or equivalent
- Associate/Assistant Professor/Senior researcher/Lecturer or equivalent
- Teacher
- Post-doc
- PhD student/Research or teaching assistant
- Technical and administrative research staff (Research coordinator or laboratory personnel)
- Other, please specify

44) Appointment

- Temporary
- Permanent

45) Where do you get your research funding? (several answers possible)

- ☐ Own institution
- ☐ National research councils
- ☐ Public or non-profit organizations
- ☐ Industry
- ☐ EU and other international funds
- ☐ Not applicable

46) Years active in research

- ○ 0-5
- ○ 6-10
- ○ 11-15
- ○ 16 or more
- ○ Not applicaple

© Copyright www.questback.com. All Rights Reserved.
